# Robotic Surgery in Guyana: First Report From the Caribbean

**DOI:** 10.7759/cureus.112271

**Published:** 2026-07-08

**Authors:** Hemraj Ramcharran, Jagnanand Ramnarine, Bibi Hussain, Mahendra Carpen, Navindranauth Rambaran, Rajendra Sukhraj, Radha Sookraj, Padmini Singh, Shamir O Cawich

**Affiliations:** 1 Surgery, Georgetown Public Hospital Corporation, Georgetown, GUY; 2 Urology, College of Medical Sciences, University of Guyana, Georgetown, GUY; 3 Obstetrics and Gynaecology, Georgetown Public Hospital Corporation, Georgetown, GUY; 4 Surgery, Port of Spain General Hospital, Port of Spain, TTO

**Keywords:** minimally invasive surgery (mis), robotic, simulation technology, surgical equity, the caribbean region

## Abstract

Although robotic surgery is widely accepted as the gold standard approach for a variety of operative procedures, the Caribbean region lacked access to surgical robotics until recently. The surgical fraternity in Guyana recognized the need for progress to robotic surgery. Herein, we report the first robotic surgery procedure performed in Guyana and highlight the obstacles and challenges faced, and the success achieved while introducing the technology in a low-resource setting.

Using the SSI Mantra 3 Robotic Surgical System (SS Innovations International Inc., Haryana, India), a multidisciplinary team carried out a procedure on a 34-year-old man with a right inguinal hernia. From the surgical command centre, the lead surgeon dissected a preperitoneal space to reduce the sac and reinforce the inguinal canal with a lightweight polypropylene mesh. The peritoneum was closed to complete a robotic trans-abdominal pre-peritoneal (TAPP) repair. To our knowledge, this was the first robotic TAPP procedure recorded in the English-speaking Caribbean.

With the support of administrators and buy-in from surgical leaders, the surgical fraternity in Guyana has seized a broader vision to modernize healthcare services, providing advanced surgical care in the Caribbean region. We demonstrated that robotic surgery is feasible and safe in the Caribbean environment, provided there is appropriate training, proctorship, and selection of robotic platforms. This report should encourage other countries in the Caribbean region to transition to robotic surgery. In our experience, the success of our programme depended on four pillars: (1) strong leadership support, (2) buy-in from the surgical community, (3) involving all specialties to maximize case volumes, and (4) selection of an affordable and effective platform.

## Introduction

Robotic surgery is widely accepted as the gold standard approach for a variety of operative procedures [1]. There are numerous benefits to using this surgical technique. Patients experience reduced morbidity, improved quality outcomes, and decreased hospital length of stay [1]. Surgeons benefit from improved ergonomics, better instrument dexterity, three-dimensional, high-resolution vision, reduced musculoskeletal strain, and tremor filtration [1-3]. Together, these advantages have propelled robotic surgery to become the gold standard approach for a variety of surgical procedures [1-5].

Over the past two decades, the surgical community in Guyana has made a concerted effort to advance minimally invasive surgery [6]. With the established momentum, the surgical fraternity in Guyana recognized the need to progress to robotic surgery. In this report, we share our experience developing a robotic service and performing our first robotic surgery procedure in a low-resource setting.

## Case presentation

A 34-year-old man with no medical illnesses presented to the surgical clinic after noticing a reducible, non-tender swelling in the right groin. Clinical examination confirmed an uncomplicated right inguinal hernia, and based on this diagnosis, the patient was scheduled for elective herniorrhaphy. The procedure was carried out on May 26, 2026, at the Georgetown Public Hospital Corporation (GPHC), where a team of local surgeons with proctors from multiple institutions used the SSI Mantra 3 Robotic Surgical System (SS Innovations International Inc., Haryana, India) to repair the right inguinal hernia.

**Figure 1:**
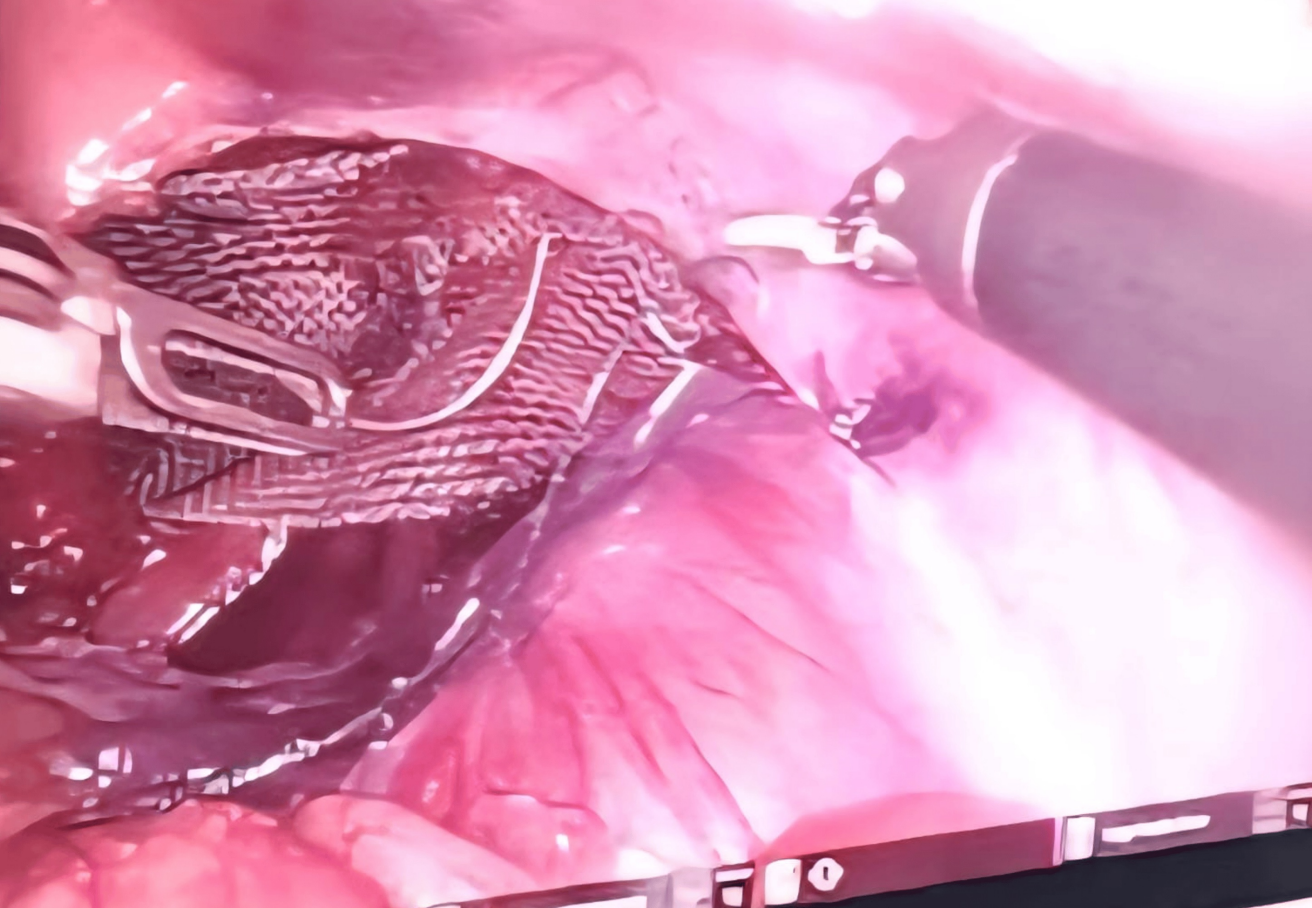
Intra-operative image: the pre-peritoneal space was created and a mesh was inserted into the pocket. The peritoneum was closed with sutures.

On the day of surgery, Hasson’s technique was used to establish a pneumoperitoneum and insert ports. The robot was subsequently docked, and the surgeon occupied the surgical command centre. Using the robotic instruments, the preperitoneal space was dissected to reduce the hernia sac and create a pocket for mesh placement. A lightweight polypropylene mesh was placed in the pre-peritoneal space and fixated with V-Loc sutures (Figure 1). The peritoneum was closed to complete the robotic trans-abdominal pre-peritoneal (TAPP) repair. This was the first robotic TAPP procedure recorded in the English-speaking Caribbean.

The patient was ambulant and tolerated diet on day 1. There were no immediate complications recorded, and the patient was discharged from the hospital after 24 hours. He was reviewed as an outpatient on day 7 and remained clinically well. Up to day 14, there was no evidence of a recurrence.

## Discussion

Despite the proven advantages, there has been a lack of access to robotic equipment in low- and middle-income countries (LMICs) due to high capital costs [1-5], unsupportive leadership [1,7], unavailability of distributors [8], and low case volumes [9,10]. An attempt to introduce the Freehand® robotic camera arm on September 15, 2021, at the Port of Spain General Hospital in Trinidad and Tobago [8] was greeted with short-lived interest that waned shortly after, largely due to unsupportive surgical leadership.

Recognizing the need for advancement in minimally invasive surgery, the surgical fraternity in Guyana engaged hospital administrators and policymakers from the Government of Guyana. Effective lobbying efforts combined with the existing data on the advantages of robotic surgery were rewarded with a memorandum of understanding for the nation and the Caribbean region to transition to robotic surgery. With leadership support at the highest level by His Excellency President Dr. Mohamed Irfaan Ali and the Minister of Health, Dr. Frank Anthony, funding was allocated to procure and install a robotic platform.

**Figure 2:**
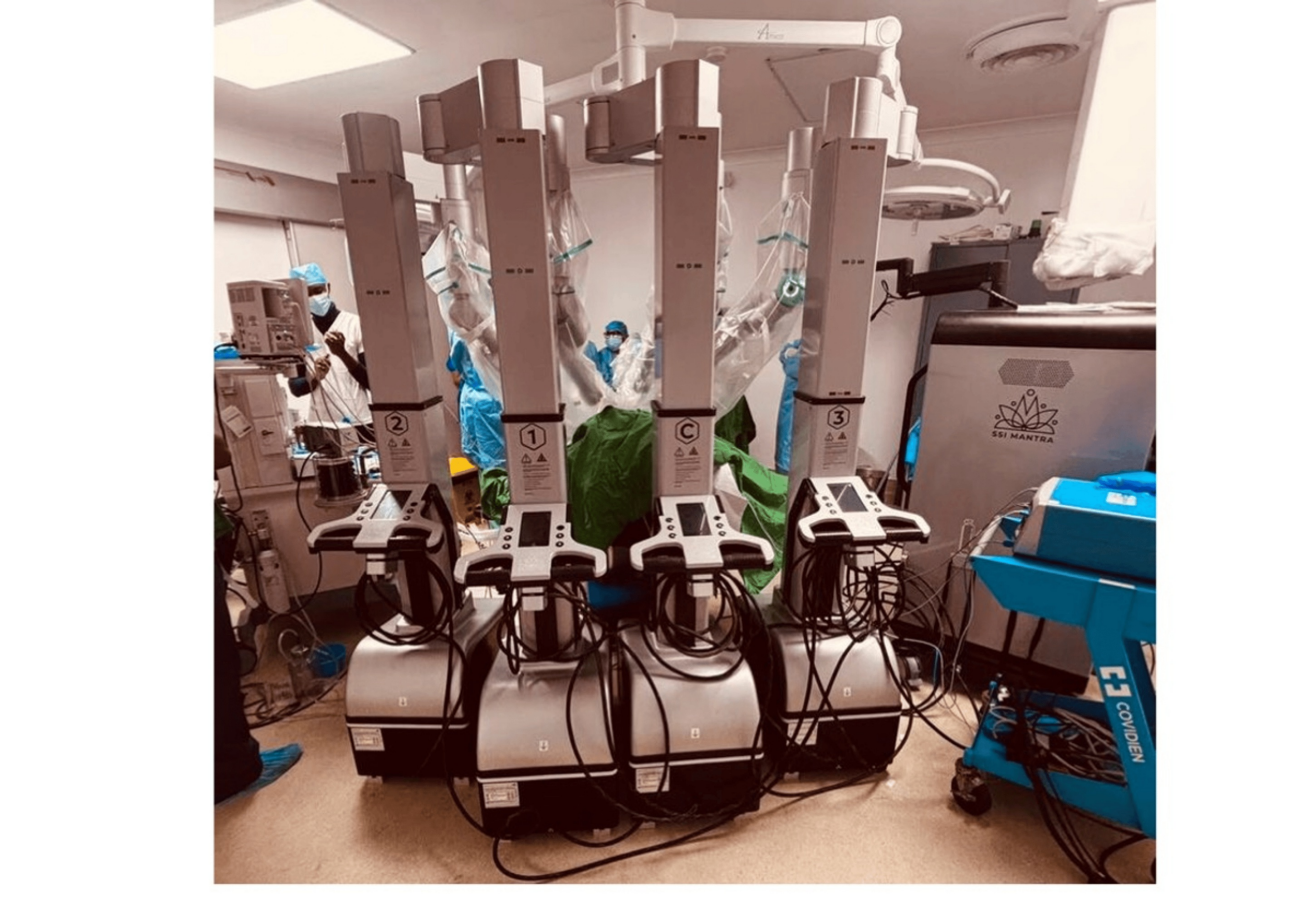
Intra-operative photograph showing four modular robotic arms docked over the patient to control varied robotic instruments.

The platform selected was the SSI Mantra 3 Robotic Surgical System created by SS Innovations International Inc. [11]. This system consists of an open-faced surgeon command center from which the surgeon receives visual feedback through 3D glasses on a 4K monitor, and controls the modular arms using hand and foot controls. Similar to the Versius (CMR Surgical Ltd., Cambridge, United Kingdom) and Hugo (Medtronic, Minneapolis, USA) systems, the SSI Mantra 3 Robot is a modular system with four independent arms that fully control robotic instruments (Figure 2). This system was chosen due to its versatility in a variety of surgical scenarios as well as its affordability. Our capital investment to acquire and install the SSI Mantra platform was approximately 35% of the cost for a da Vinci® platform by Intuitive Surgical, Inc. (Sunnyvale, USA) [1,12]. This made it suitable for our resource-constrained settings.

Once the desired platform was identified, a collaboration occurred between the Government of Guyana, the Ministry of Health, Georgetown Public Hospital Corporation, SS Innovations International Inc., and the regional distributor, New Guyana Pharmaceutical Corporation (GPC). Funding was allocated to this new initiative, and the Government of Guyana purchased the SSI Mantra 3 Robotic Surgical System as part of a national initiative to strengthen tertiary healthcare services. New GPC served as the local technical partner responsible for equipment support, maintenance, consumables, software upgrades, and ongoing technical development.

During the period when the equipment was sent by freight to Guyana, other developments occurred simultaneously. A robotic surgical team was assembled by the surgical leaders, and they traveled to the supplier in India for training on a simulator for two weeks, followed by experience scrubbing into live cases. A deliberate decision was taken for the robotic surgery team to comprise surgeons from multiple disciplines, as well as relevant supporting staff. This was done to maintain case volumes to ensure appropriate usage of the platform by all specialties.

Following appropriate training, the SSI Manta 3 platform was installed, and the first robotic TAPP procedure was performed on May 26, 2026. This was 19 years after the laparoscopic TAPP technique was introduced to the Caribbean in 2005 [13], and 12 years after the first laparoscopic TAPP procedure was performed in Guyana on June 6, 2014 [6]. It is clear that the surgical fraternity in Guyana has made rapid progress to move from the first laparoscopic cholecystectomy in Guyana in 2002 [6] to robotic operations within two decades. Since the index TAPP repair, a variety of general surgical, urologic, and gynaecologic cases have been performed, including cholecystectomies, appendectomies, gastric bypass, salpingectomies, ovarian cystectomy, varicocelectomy, and renal cyst decortication.

There are added advantages since the robotic platform incorporates integrated data management systems capable of securely storing operative information and procedural metrics. This facilitates data collection for clinical outcomes research, medical education, and the development of evidence-based protocols tailored to the Guyanese population. Over time, we expect to develop a national robotic surgery registry, generating valuable data from Guyana and contributing to the regional and international surgical literature.

We believe that this experience should encourage LMICs in the Caribbean region to transition to robotic surgery. In our experience, the success of our programme depended on four pillars: (1) strong leadership support, (2) buy-in from the surgical community, (3) involving all specialties to maximize case volumes, and (4) selection of an affordable and effective platform.

## Conclusions

With the support of administrators and buy-in from surgical leaders, the surgical fraternity in Guyana has embraced a broader vision to modernize healthcare services, providing advanced surgical care in the Caribbean region. We demonstrated that robotic surgery is feasible and safe in the Caribbean environment, provided there is appropriate training, proctorship, and selection of robotic platforms. This report should also motivate surgeons in the region to support the development of robotics within the Caribbean.
